# Serum Galanin Levels in Young Healthy Lean and Obese Non-Diabetic Men during an Oral Glucose Tolerance Test

**DOI:** 10.1038/srep31661

**Published:** 2016-08-23

**Authors:** Héctor Fabio Sandoval-Alzate, Yessica Agudelo-Zapata, Angélica María González-Clavijo, Natalia E. Poveda, Cristian Felipe Espinel-Pachón, Jorge Augusto Escamilla-Castro, Heidy Lorena Márquez-Julio, Hernando Alvarado-Quintero, Fabián Guillermo Rojas-Rodríguez, Juan Manuel Arteaga-Díaz, Javier Hernando Eslava-Schmalbach, Maria Fernanda Garcés-Gutiérrez, Maria Vrontakis, Justo P. Castaño, Raul M. Luque, Carlos Diéguez, Rubén Nogueiras, Jorge E. Caminos

**Affiliations:** 1 Department of Internal Medicine–Division of Endocrinology, School of Medicine, Universidad Nacional de Colombia, Bogotá, Colombia; 2 Department of Physiology, School of Medicine, Universidad Nacional de Colombia Bogotá, Colombia; 3 Institute of Clinical Investigations, School of Medicine, Universidad Nacional de Colombia, Bogotá, Colombia; 4 Department of Human Anatomy & Cell Science, Faculty of Medicine, University of Manitoba, Winnipeg, Manitoba, Canada; 5 Instituto Maimónides de Investigación Biomédica de Córdoba (IMIBIC), Córdoba, Spain; 6 Department of Cell Biology, Physiology, and Immunology, University of Córdoba, Córdoba, Spain; 7 Hospital Universitario Reina Sofía, Córdoba, Spain; 8 Department of Physiology (CIMUS), School of Medicine, University of Santiago de Compostela, Santiago de Compostela, Spain; 9 Instituto de Investigaciones Sanitarias (IDIS), University of Santiago de Compostela, Santiago de Compostela, Spain; 10 CIBER Fisiopatología de la Obesidad y Nutrición (CIBERobn), Spain

## Abstract

Galanin (GAL) is a neuropeptide involved in the homeostasis of energy metabolism. The objective of this study was to investigate the serum levels of GAL during an oral glucose tolerance test (OGTT) in lean and obese young men. This cross-sectional study included 30 obese non-diabetic young men (median 22 years; mean BMI 37 kg/m^2^) and 30 healthy lean men (median 23 years; mean BMI 22 kg/m^2^). Serum GAL was determined during OGTT. The results of this study include that serum GAL levels showed a reduction during OGTT compared with basal levels in the lean subjects group. Conversely, serum GAL levels increased significantly during OGTT in obese subjects. Serum GAL levels were also higher in obese non-diabetic men compared with lean subjects during fasting and in every period of the OGTT (p < 0.001). Serum GAL levels were positively correlated with BMI, total fat, visceral fat, HOMA–IR, total cholesterol, triglycerides and Leptin. A multiple regression analysis revealed that serum insulin levels at 30, 60 and 120 minutes during the OGTT is the most predictive variable for serum GAL levels (p < 0.001). In conclusion, serum GAL levels are significantly higher in the obese group compared with lean subjects during an OGTT.

Galanin (GAL) is a 29/30 amino acid peptide that belongs to the GAL peptide family^1^ and is widely distributed in the central and peripheral nervous system, adipose tissue, skeletal muscle, and the enteric tract of many mammals^2^. Although GAL is involved in a wide variety of physiological functions^2^, it is mainly known for its important role in energy balance, glucose and insulin metabolism^3^,^4^,^5^.

Several reports in rodents have supported the role of GAL in energy and glucose homeostasis. The central injection of GAL induces feeding in rats^6^ and this effect is blocked by the administration of the GAL antagonists, C7 and M40^7^. In cultured L and K cells from mice intestine it was observed that GAL and M617, a selective agonist of the receptor GALR1, inhibit the secretion of both glucose-dependent peptide (GIP) and the glucagon-like peptide 1 (GLP-1)^8^. In accordance with these pharmacological studies, transgenic mice with high levels of endogenous GAL develop obesity and alterations in lipid metabolism. Lately, GAL appears to have a different role according to the metabolic context of the individual, tending to improve insulin resistance in scenarios such as type 2 diabetes mellitus^3^,^9^,^10^,^11^.

Human studies have shown some controversial results, since some reports found higher GAL levels in obese individuals when compared to healthy control women, whereas others failed to detect significant differences^12^. Also, plasma GAL levels were elevated in obese young women in comparison with normally menstruating women^13^. Additionally, recent studies show significantly high serum GAL levels in pregnant women with gestational diabetes^14^,^15^,^16^, but no differences in GAL levels were detected in neonates born to gestational diabetic mothers, neonates with intrauterine growth restriction and healthy neonates^17^. GAL levels were also increased in type 1 and type 2 diabetes^3^,^14^,^16^,^18^,^19^, positively correlated with the blood glucose level^3^,^20^, and with hemoglobin A1c content among type 1 diabetes mellitus patients^18^. However, the intravenous infusion of GAL showed that plasma glucose and insulin levels remained unchanged during a glucose tolerance test in healthy male volunteers^21^ or diabetic and non-diabetic patients with acromegaly^22^. Therefore, the existence of GAL resistance in subjects with type 2 diabetes mellitus has been proposed^23^. In the present study, we analyzed serum GAL levels in a group of healthy lean young men and obese non-diabetic young men during an oral glucose tolerance test (OGTT) to further explore the new emerging concept of GAL resistance in obese subjects. Additionally, these GAL levels were correlated with anthropometric, biochemical and hormonal parameters.

## Results

All clinical, anthropometric, biochemical and hormonal parameters of the subjects are showed in Table 1. No statistical differences exist between healthy lean men and obese non-diabetic men in terms of age, lean: 23 (21–26) years, and obese: 22 (21–26) years; p > 0.05). Conversely, anthropometric, biochemical and hormonal parameters were significantly different between groups (Table 1).

Significant differences between healthy lean subjects and obese non-diabetic subjects were found for BMI (22.18 ± 1.75 Kg/m^2^ vs. 37.83 ± 4.92 Kg/m^2^; p < 0.001) ; total body fat (percent of total body composition) (21.14 ± 4.46% vs. 45.07 ± 3.70%; p < 0.001); visceral fat (percent of total body fat) (28.93 ± 6.68% vs. 55.66 ± 3.16%; p < 0.001); waist circumference (78.20 ± 4.45 cm vs. 110.92 ± 8.41 cm; p < 0.001); and gynoid (peripheral) fat (27.30 ± 4.56% vs. 47.32 ± 3.78%; p < 0.001) (Table 1).

Serum glucose levels determined during OGTT were statistically different between healthy lean subjects and obese non-diabetic subjects only at 60 and 120 minutes: 60 min (86.87 ± 19.36 mg/dL vs. 112.40 ± 31.28 mg/dL; p < 0.001) and 120 min (75.17 ± 13.75 mg/dL vs. 90.77 ± 27.63 mg/dL; p = 0.008) (Table 1, Supplementary Figure 1). In addition, obese subjects have significantly higher serum insulin levels compared to lean subjects during OGTT: 0 min (7.93 ± 3.08 μUI/mL vs. 28.06 ± 12.77 μUI/mL ;p < 0.001); 30 min [74.10 (41.58–109.40) μUI/mL vs. 213.70 (128.70–262.20) μUI/mL; p < 0.001]; 60 minutes [50.50 (32.5–73.43) μUI/mL vs. 134.60 (95.25–184.40) μUI/mL; p < 0.001]; and 120 minutes [23.30 (12.88–35.92) μUI/mL vs. 74.30 (34.22–119.80) μUI/mL; p < 0.001] (Table 1, Supplementary Figure 1). Furthermore, HOMA-IR was also significantly different between lean and obese subjects [1.56 (1.01–2.03) vs. 5.77 (3.99–7.70); p < 0.001] (Table 1, Supplementary Figure 2).

Additionally, the lipid profile also showed significant differences between healthy lean and obese non-diabetic groups: total cholesterol (165.73 ± 25.28 mg/dL vs. 189.30 ± 27.65 mg/dL; p < 0.001); cHDL [47.0 (40.0–52.75) mg/dL vs. 39.0 (35.0–43.0) mg/dL; p < 0.001]; cVLDL [16.60 (13.70–21.60) mg/dL vs. 30.90 (21.70–44.05) mg/dL; p < 0.001] and triglycerides [83.0 (68.50–109.80) mg/dL vs. 158.50 (116.80–220.20) mg/dL; p < 0.001] (Table 1, Supplementary Figure 3).

Fasting serum leptin levels were significantly lower in lean compared to obese subjects group [7389.0 (6722.0–7882.0) pg/mL vs. 21640.0 (17090.0–32110.0) pg/mL; p < 0.001] (Table 1). Conversely, fasting serum adiponectin levels were significantly increased in lean compared to obese subjects group (15.09 ± 1.92 μg/mL vs. 13.29 ± 1.96 μg/mL; p < 0.001) (Table 1).

**Figure 1:**
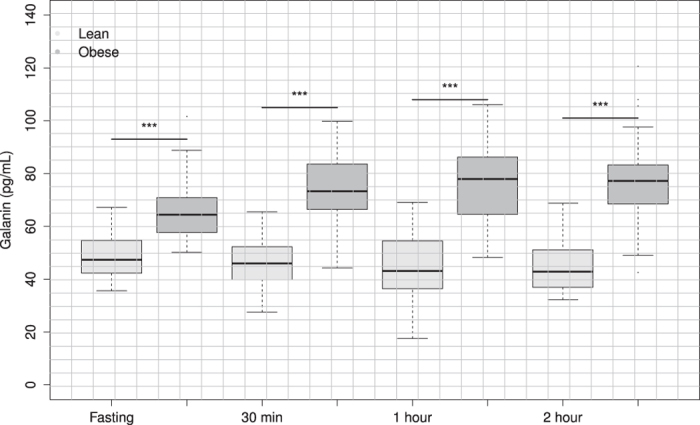
Comparison of serum GAL levels between healthy lean men and obese men at different times. The box-and-whisker plots represent the serum GAL levels during an oral glucose tolerance test (fasting, 30, 60 and 120 minutes) analyzed in healthy, lean and young men, and obese non-diabetic men. Statistical significance is shown in brackets. Points represent outliers.

GAL serum levels were statistically different between control and obese subjects at each of the OGTT time points: 0 min [47.47 (42.13–54.70) pg/mL vs. 64.46 (57.75–70.32) pg/mL; p < 0.001); 30 min (46.18 ± 9.79 pg/mL vs. 73.48 ± 13.97 pg/mL; p < 0.001), 60 min (43.57 ± 13.34 pg/mL vs. 75.25 ± 14.35 pg/mL; p < 0.001); and 120 min [42.68 (36.95–50.41) pg/mL vs. 77.18 (68.62–83.25) pg/mL; p < 0.001] (Table 1, Fig. 1).

**Figure 2:**
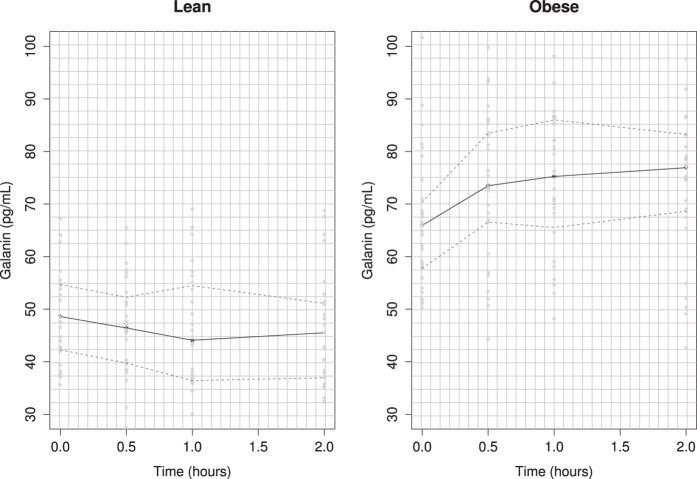
Serum GAL levels in healthy lean men and obese men during an oral glucose tolerance test (OGTT). The curves show GAL levels measured at four points in time (fasting, 30, 60 and 120 minutes), in both healthy, lean and young men, and obese non-diabetic men.

Additionally, obese subjects had lower basal GAL serum levels than those observed at 30, 60, and 120 min post-oral glucose load. Conversely, GAL serum levels in lean subjects decreased significantly from basal to 60 min during OGTT (p < 0.05) and returned to the fasting level up to 120 min after glucose (Fig. 2).

**Figure 3:**
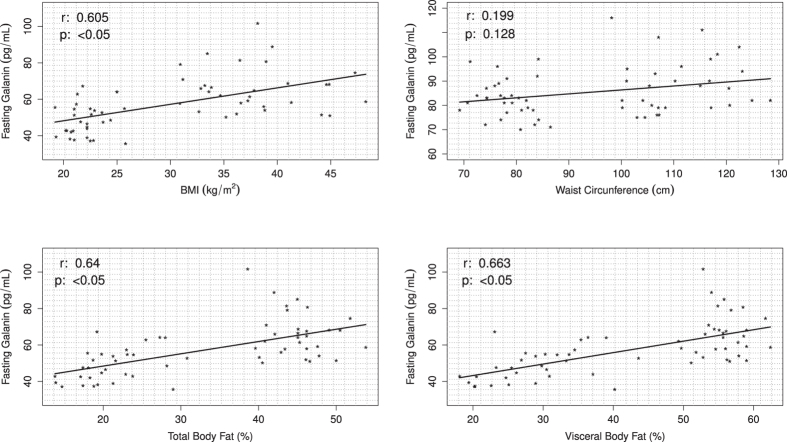
Scatterplots of body composition parameters correlated with serum fasting GAL levels in young, lean and obese non-diabetic men. Positive correlation between BMI (Body Mass Index), waist circumference, total body fat, visceral body fat and fasting GAL levels in a group of 60 young, lean and obese non-diabetic men.

**Figure 4:**
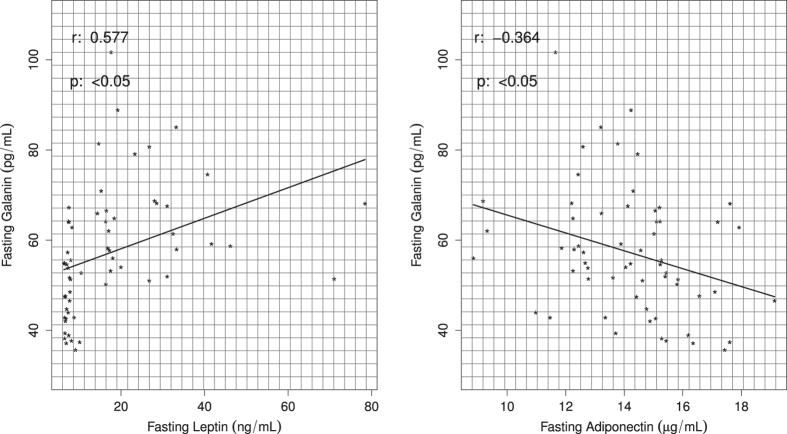
Scatterplots of leptin and adiponectin serum levels correlated with fasting GAL levels in young, lean and obese non-diabetic men. Positive correlation between fasting leptin and fasting GAL levels. Negative correlation between fasting adiponectin and fasting GAL levels, in a group of 60 young, lean and obese non-diabetic men.

The correlation analysis between GAL serum levels and the others parameters were performed in both lean and obese subjects. Fasting serum GAL levels were positively correlated with BMI (r = 0.605; p < 0.05), total fat % (r = 0.640; p < 0.05), visceral fat % (r = 0.663; p < 0.05), HOMA–IR (r = 0.648; p < 0.05), total cholesterol (r = 0.337, p < 0.05), triglycerides (r = 0.466; p < 0.05) and leptin (r = 0.577; p = <0.05) (Fig. 3, Supplementary Figure 4, Supplementary Figure 5, Fig. 4, Table 2). Also, fasting serum GAL levels were negatively correlated with adiponectin levels (r = −0.364; p = <0.05) (Fig. 4, Table 2). There were no significant correlation between serum GAL levels and cHDL (r = −0.165; p = 0.221), cLDL levels (r = 0.21; p = 0.116) and waist circumference (r = 0.199; p = 0.128) (Supplementary Figure 5, Table 2). A multiple regression analysis revealed that serum insulin levels at 30, 60 and 120 minutes during the OGTT is the most predictive variable for serum GAL levels when adjusting for the other variables (p < 0.001) (Supplementary Table 1).

**Figure 5:**
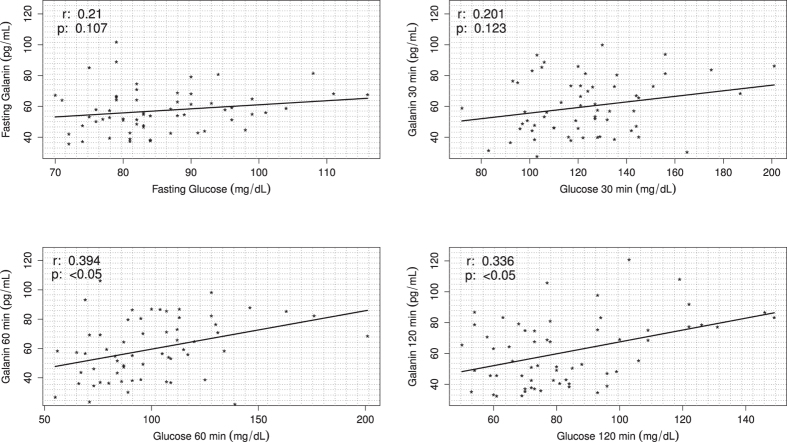
Scatterplots of an oral glucose tolerance test (OGTT) correlated with GAL levels in young, lean and obese non-diabetic men. Positive correlations between glucose and GAL levels analyzed at four points in time (fasting, 30, 60 and 120 minutes) in a group of 60 young, lean and obese non-diabetic men.

**Figure 6:**
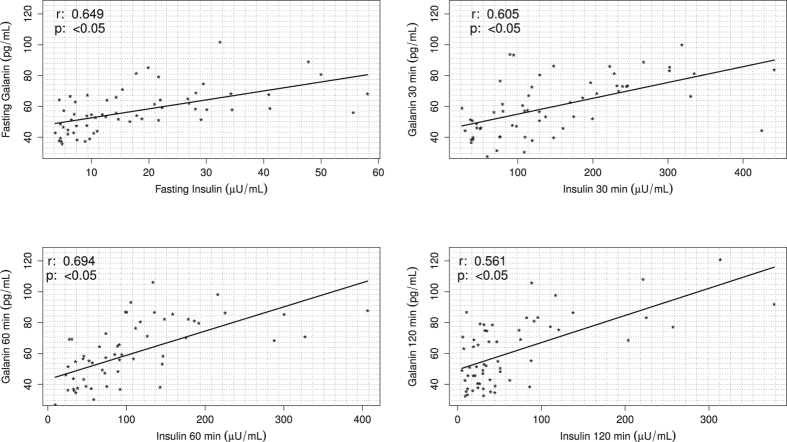
Scatterplots of insulin levels correlated with GAL levels in in young, lean and obese non-diabetic men during an oral glucose tolerance test (OGTT). Positive correlations between insulin and GAL levels analyzed at four points in time (fasting, 30, 60 and 120 minutes) in a group of 60 young, lean and obese non-diabetic men.

Finally, serum GAL levels were positively correlated with, glucose at 60 min (r = 0.394; p = <0.05), glucose at 120 min (r = 0.366; p = <0.05), fasting insulin (r = 0.649; p < 0.05), insulin at 30 min (r = 0.605; p < 0.05), insulin at 60 min (r = 0.694; p < 0.05) and insulin at 120 min (r = 0.561; p < 0.05) (Figs 5 and 6, Table 2).

## Discussion

The present study demonstrates that serum GAL levels in obese non-diabetic subjects are significantly higher compared to healthy lean subjects at basal and at each of the post oral glucose load time points 30, 60, and 120 min. Also, serum GAL levels are positively correlated with HOMA-IR, total cholesterol, triglycerides, and insulin levels at all of the OGTT time points. In addition, a similar positive correlation is observed between serum GAL levels and glucose levels at 60 and 120 minutes during OGTT. Anthropometric parameters such as BMI, total body fat and visceral fat were also positively correlated with serum GAL levels. Additionally, a multiple regression analysis revealed that insulin was the most predictive determinant of serum GAL levels. Finally, we show that serum GAL levels correlated positively with leptin levels, and negatively with serum adiponectin levels.

Recent studies demonstrated that GAL serum levels are closely related with insulin sensitivity in rat skeletal muscle and adipose tissue^24^. It has also been demonstrated that GAL contributes to the regulation of glucose homeostasis and carbohydrate metabolism in peripheral tissues^4^,^5^,^11^,^24^,^25^,^26^,^27^. GAL appears to have a different role in the scenario of insulin resistance. In GAL gene *knock out* rats, an alteration was demonstrated in the carbohydrate metabolism^9^, whereas in transgenic mice homozygous for the GAL gene a reduction in insulin resistance and an increase in lipid metabolism and carbohydrate was demonstrated^10^. Many studies have demonstrated a positive correlation between GAL and glucose levels, which leads to postulate that elevated GAL serum levels are a consequence of elevated glucose serum levels^3^,^16^. Zhang *et al*. showed a significant positive correlation between GAL and HOMA-IR, but no correlation with serum insulin levels^15^. Fang *et al*. proposed that elevated GAL levels in diabetic patients could be related to a GAL receptor signaling compensation process^23^,^24^. Thus, our findings showing the coexistence of increased GAL levels and insulin resistance are compatible with the existence of a state of GAL resistance in obese subjects. Alternatively, they could be viewed as a compensatory phenomenon to defend glucose homeostasis in obesity.

Zhang *et al*. demonstrated that plasma GAL levels were significantly higher in patients with gestational diabetes, compared to plasma GAL levels of pregnant women with normal glucose tolerance^15^. Additionally, these authors reported that plasma GAL levels are positively correlated with BMI and fasting plasma glucose in the group of women who developed gestational diabetes^15^. On the other hand, and contrary to our results, Legakis *et al*. showed that serum GAL levels increase significantly by 30 min in healthy individuals during OGTT, and return to baseline levels at 180 min^17^. Contrary to the group studied by Legakis *et al*. (48 ± 3.56 years of age, and BMI 27 ± 0.5 kg/m2), we have enrolled younger lean [23 (21–26) years of age and BMI 22.18 ± 1.75 kg/m^2^] and obese [22 (21–26) years of age and BMI 37.83 ± 4.92 kg/m^2^] subjects. Additionally, the sample size of the former study group (four men and seven women) limits the possibility to establish statistical correlations. In accordance with Legakis *et al*., the present study shows a positive correlation with HOMA-IR and serum GAL levels^17^. Additionally, our study did not show significant differences in fasting glucose levels between obese and lean subjects, and neither showed any significant correlation between fasting glucose and serum GAL levels. On the other hand, glucose levels are significantly correlated with GAL levels at 30 min, 60 min, and 120 min during OGTT, when the serum glucose levels are significantly different between obese and lean subjects. Also, in the present study, insulin levels at fasting and during the OGTT were significantly different between obese and lean subjects. There was also a statistically significant and positive relationship between insulin and GAL levels at fasting and during the OGTT. Thus, it is possible to hypothesize that elevated insulin levels might play an important role in regulating serum GAL levels and a possible synergistic relationship with glucose levels is needed to induce serum GAL level elevation in patients with insulin resistance, as observed in the present study and previous studies^11^.

Preliminary studies in humans have demonstrated that GAL intravenous infusion inhibits postprandial glucose and insulin levels^28^. A statistically positive relationship between GAL and fasting glucose in type 2 diabetes mellitus patients was shown by Legakis *et al*.^20^, but such relationship was not found in the present study, possibly because our subjects were not diabetic. Throughout different studies in animal and human models, the type 2 diabetes mellitus phenotype has been characterized by obesity, hyperglycemia, hyperinsulinemia, insulin resistance, elevated GAL levels and a reduced activity of the GAL receptor^29^. GAL increases fat deposition and contributes to the development of obesity by favoring glucose utilization over fat utilization^1^. It has been previously shown that GAL mRNA expression is up-regulated in rat adipose tissue after fasting^30^. Furthermore, GAL-transgenic mice showed an increase in body weight, total cholesterol, triglycerides, visceral adiposity and insulin levels^10^. It has also been demonstrated that GAL serum levels are elevated in obese patients, compared to non-obese control subjects^31^.Young obese women showed elevated GAL serum levels compared to healthy controls, which suggests that GAL is related with obesity and overfeeding^32^. All these results leads to the conclusion that increased body weight is related to elevated levels of GAL. Thus, previous studies are in concordance with our results that GAL has a statistically significant and positive relationship with BMI, cholesterol and triglycerides levels. Then, it is possible to propose that GAL could be contributing to the increase of adiposity in those subjects.

It has been demonstrated that the intracerebroventricular (ICV) administration of Gal increases deposits in adipose tissue between un 30% a 40%^33^. Other studies carried out in male albino *Sprague Dawley* rats have shown that diets rich in fatty acids lead to hyperglycemia, an increase in adiposity, and a significant increase in the expression and production of GAL peptide in the central nervous system^34^. Yun *et al*., demonstrated an increase in phosphofructokinase activity and a reduction in β-hydroxyacyl-CoA dehydrogenase activity in muscle, which implies an increase in the capacity to metabolize carbohydrates and a reduction in the fatty acid oxidation in the muscle^33^.

GAL resistance is an emergent concept and is defined as the presence of elevated serum GAL levels in order to lead serum glycemic levels to normal values^23^. This is a concept similar to those of insulin resistance, in which there is a compensatory rise in serum insulin levels to increase GLUT 4 translocation to the cell membrane in skeletal muscle and adipose tissue. Bu *et al*., demonstrated that the coadministration of GAL and insulin reduced insulin resistance through an increase of GLUT4 translocation to the cellular membrane in rat myocytes with type 2 diabetes mellitus. This effect was associated with the upregulation of Protein Kinase C (PKC) and Akt substrate of 160 Kd (AS 160), molecules in the down-stream signaling pathway of the insulin receptor that could be shared with those of GAL signaling^11^. Additionally, Liang *et al*., discovered the increase in the translocation of GLUT4 transporters in adipocytes of diabetic rats when the liberation of GAL was induced through exercise. This same study found an inhibitory effect on the expression of GLUT 4 mRNA when using GALR antagonists, M35^35^. Finally, He *et al*. also demonstrated a similar effect described by Lang *et al*. in adipose tissue, but this time in muscle cells of rats with type 2 diabetes mellitus^4^. These studies demonstrate the close relationship of the signaling pathways shared by GAL and insulin. This may support the independent association between insulin and GAL described in this study, reinforcing the concept of GAL resistance.

GAL induces GLUT4 translocation^23^,which may converge with other adipocytokines pathways, such as leptin and adiponectin^36^. Previous studies described that leptin exerts similar effects of those of insulin in the translocation of GLUT4, particularly in skeletal muscle^37^. Baranoskowa *et al*., described both hyperleptinemia and hypergalaninemia in obese (non-pregnant) women^31^. Additionally, hypoadiponectinemia observed in obese patients is the result of the increase of pro-inflammatory cytokines, such as Tumor Necrosis Factor alpha (TNF-α) and Interleukin 6 (IL-6), involved in the silencing of the adiponectin gene^38^. The above cited proinflammatory condition enhances the ubiquitinization and accelerates the degradation of Insulin Receptor Substrate 1 (IRS-1) needed for the normal function of the insulin signaling pathway^39^.

Like leptin, adiponectin (an anti-inflammatory adipocytokine) improves insulin sensitivity and increases GLUT-4 translocation^37^. The present study showed that serum GAL and leptin levels were positively correlated, whereas GAL and adiponectin levels showed a negative correlation. This might help to explain the putative role of GAL and these adipocytokines in obesity.

In conclusion, the present study demonstrates: 1) serum GAL levels decrease during the OGTT in lean subjects, 2) serum GAL levels increase significantly during OGTT in obese subjects, 3) serum GAL levels are significantly elevated at fasting and throughout the OGTT in obese men, compared with lean controls, 4) GAL is positively correlated with leptin, BMI, total body fat, visceral fat, HOMA-IR, insulin, glucose, total cholesterol and triglycerides, and correlates negatively with adiponectin and finally, 5) serum insulin levels may be an important predictor of serum GAL levels. According to our results, it is possible that high serum GAL levels are related to insulin resistance and body weight increase in obese non-diabetic subjects, and these results support the emerging concept of GAL resistance in obese humans and additional studies are required to confirm these results.

## Materials and Methods

### Patients

The protocol of this study was approved by the Ethical Committee of the School of Medicine of the Universidad Nacional de Colombia. All experiments were performed in accordance with relevant guidelines and regulations. The procedures were clearly explained and all research participants gave their written informed consent for the study before participation. This cross-sectional study was conducted during 2015.

The study group included 30 obese non-diabetic young men (24.12 ± 3.96 years of age; mean BMI 38.54 ± 4.57 kg/m^2^) and 30 age-matched lean men (24.68 ± 3.56 years of age; mean body mass index (BMI) 22.13 ± 1.85 kg/m^2^). Physicians and nutritionists examined all participants and the descriptions of the clinical evaluation and anthropometric characteristics are shown in Table 1.

Body composition was determined by Dual-Energy X-ray absorptiometry (DXA) (GE Lunar Prodigy Advance). Obesity was defined as a BMI >30 kg/m^2^, according to the World Health Organization and the International Obesity Task Force classification criteria^40^. We excluded subjects with a diagnosis of diabetes mellitus, a diagnosis of any chronic disease (kidney failure, coronary heart disease, thyroid disease, among others) or patients who underwent bypass or any other bariatric surgery. We also excluded subjects taking any medication such as levothyroxine, metformin, or steroids, within the last 12 months.

### Methods

All subjects were given a 75 g/300 ml oral glucose solution at 07:00 am, after an overnight fast. Blood samples were withdrawn in the fasting state (at 0 minutes) and 30, 60 and 120 minutes during OGTT. Serum samples were separated by centrifugation at 1000 g for 15 minutes and stored at −80 °C until assay. Serum triglycerides, total cholesterol, High Density Cholesterol (cHDL), Low Density Cholesterol (cLDL), Very Low Density Cholesterol (cVLDL), glucose and insulin were measured as previously described^41^. The Homeostasis Model Assessment-Insulin Resistance index (HOMA-IR) was calculated in all subjects as described by Matthews *et al*.^42^.

Human serum GAL was measured using a commercially available ELISA kit (Catalog Number CEB084Hu-Wuhan USCN Business Co., Ltd). The intra and inter assay coefficients of variation (CVs) were <10% and <12% respectively. Additionally, human serum leptin concentrations were measured with a commercially available human ELISA kit (Catalog Number KAC2281 - Thermo Fisher Scientific Inc) with an intra and inter assay coefficients of variation (CVs) of <3.9% and <5.3% respectively. Finally, human adiponectin serum levels were measured with a commercially available human ELISA KIT (Catalog Number KHP0041 - Thermo Fisher Scientific Inc) and the intra and inter assay coefficients of variation (CVs) were <3.8% and <5.5% respectively. All samples were analyzed in duplicate, and the mean value of the two measurements was reported.

### Statistical analyses

For the statistical analyses, R statistical software was utilized (version 3.2.2). The normal distribution of data was verified using the Shapiro test. The results with normal distribution are shown as mean ± SD (standard deviation) and data non-normally distributed are shown as median and interquartile range (IQR). Statistical significance between two groups was determined by T test if both variables had a normal distribution, otherwise a Student’s Wilcoxon signed-rank test was utilized. We performed a univariate analysis to examine the correlation between the serum GAL levels, biochemical and anthropometric variables, using a Pearson’s or Spearman’s correlation coefficient according to the distribution of data; the first was applied to normally distributed data and the second one to non-normally distributed data. A multiple regression analysis was performed in order to evaluate the independent relationship between fasting serum GAL levels with respect to all the other variables. At the different time points of the OGTT (at 30, 60, and 120 minutes), a multiple regression analysis was performed evaluating insulin and glucose levels in comparison to serum GAL levels. All of these values are shown in Supplementary Table 1. The results were considered statistically significant in all analyses if p < 0.05.

## Additional Information

**How to cite this article**: Sandoval-Alzate, H. F. *et al*. Serum Galanin Levels in Young Healthy Lean and Obese Non-Diabetic Men during an Oral Glucose Tolerance Test. *Sci. Rep.* **6**, 31661; doi: 10.1038/srep31661 (2016).

## Supplementary Material

Supplementary Information

## Figures and Tables

**Table 1:** Anthropometric, clinical, biochemical and serum parameters of healthy lean and obese men.

Variable	Healthy lean men (n = 30)	Obese men (n = 30)	p-value
Age, years (median-IQR)	23 (21–26)	22 (21–26)	0.49
BMI, Kg/m^2^ (mean ± SD)	22.18 (±1.75)	37.83 (±4.92)	<0.001
Waist circumference, cm (mean ± SD)	78.20 (±4.45)	110.92 (±8.41)	<0.001
Total fat, % (mean±SD)	21.14 (±4.46)	45.07 (±3.70)	<0.001
Visceral fat (android), % (mean ± SD)	28.93 (±6.68)	55.66 (±3.16)	<0.001
Gynoid fat, % (mean±SD)	27.30 (±4.56)	47.32 (±3.78)	<0.001
Systolic BP, mmHg (mean ± SD)	111.90 (±10.73)	129.43 (±12.12)	<0.001
Diastolic BP, mmHg (mean ± SD)	70.27 (±7.90)	85.03 (±10.45)	<0.001
Mean BP, mmHg (mean±SD)	82.24 (±6.98)	100.07 (±9.70)	<0.001
Fasting glucose, mg/dL (median-IQR)	82.50 (78.0–86.25)	87.5 (79.25–94.75)	0.058
Glucose 30 min, mg/dL (median-IQR)	116.50 (102.20–128.80)	125.0 (108.80–142.80)	0.101
Glucose 60 min, mg/dL (mean ± SD)	86.87 (±19.36)	112.40 (±31.28)	<0.001
Glucose 120 min, mg/dL (mean±SD)	75.17 (±13.75)	90.77 (±27.63)	0.008
Fasting insulin, μUI/mL (mean ± SD)	7.93 (±3.08)	28.06 (±12.77)	<0.001
Insulin 30 min, μUI/mL (median-IQR)	74.10 (41.58–109.40)	213.70 (128.7–262.2)	<0.001
Insulin 60 min, μUI/mL (median- IQR)	50.50 (32.55–73.43)	134.60 (95.25–184.40)	<0.001
Insulin 120 min, μUI/mL (median-IQR)	23.30 (12.88–35.92)	74.30 (34.22–119.80)	<0.001
HOMA-IR (median-IQR)	1.56 (1.01–2.03)	5.77 (3.99–7.70)	<0.001
Total cholesterol, mg/dL (mean ± SD)	165.73 (±25.28)	189.30 (±27.65)	0.001
HDL-cholesterol, mg/dL (median-IQR)	47.0 (40.0–52.75)	39.0 (35.0–43.0)	<0.001
LDL-cholesterol, mg/dL (mean ± SD)	100.77 (±22.70)	113.43 (±27.0)	0.054
VLDL-cholesterol, mg/dL (median-IQR)	16.60 (13.70–21.60)	30.90 (21.70–44.05)	<0.001
Triglycerides, mg/dL (median-IQR)	83.0 (68.50–109.80)	158.50 (116.80–220.20)	<0.001
Fasting galanin, pg/mL (median-IQR)	47.47 (42.13–54.70)	64.46 (57.75–70.32)	<0.001
Galanin 30 min, pg/mL (mean ± SD)	46.18 (±9.79)	73.48 (±13.97)	<0.001
Galanin 60 min, pg/mL (mean ± SD)	43.57 (±13.34)	75.25 (±14.35)	<0.001
Galanin 120 min, pg/mL (median-IQR)	42.68 (36.95–50.41)	77.18 (68.62–83.25)	<0.001
Leptin, pg/mL (median-IQR)	7389.0 (6722.0–7882.0)	21640.0 (17090.0–32110.0)	<0.001
Adiponectin, μg/mL (mean ± SD)	15.09 (±1.92)	13.29 (±1.96)	0.001

Normally distributed data are presented as mean ± SD and non-normally distribute data are presented as median (IQR). If the variables in both groups are normally distributed, a T test was performed. If one or both of the variables are non-normally distributed, a non-parametric analysis was performed using Wilcoxon signed-rank test. A p-value <0.05 was considered statistically significant. HOMA (Homeostasis Model Assessment).

**Table 2:** Correlation between GAL and anthropometric, biochemical and serum parameters.

Variable	r	p-value
**Fasting GAL**		
BMI, Kg/m^2^ *	0.605	<0.05
Total fat, % *	0.640	<0.05
Waist circumference, cm *	0.199	0.128
Visceral fat (android), % *	0.663	<0.05
Fasting Glucose, mg/dL *	0.210	0.107
Fasting insulin, μUI/mL **	0.649	<0.05
HOMA-IR *	0.648	<0.05
Total cholesterol, mg/dL *	0.337	<0.05
HDL-cholesterol, mg/dL *	−0.230	0.077
LDL-cholesterol, mg/dL *	0.231	0.076
Triglycerides, mg/dL *	0.466	<0.05
Leptin, pg/mL *	0.577	<0.05
Adiponectin, μg/mL *	−0.364	<0.05
**GAL 30 min**		
Glucose 30 min, mg/dL **	0.201	0.123
Insulin 30 min, μUI/mL **	0.605	<0.05
**GAL 60 min**		
Glucose 60 min, mg/dL **	0.394	<0.05
Insulin 60 min, μUI/mL **	0.694	<0.05
**GAL 120 min**		
Glucose 120 min, mg/dL **	0.336	<0.05

Univariate analysis using Pearson’s or Spearman’s correlation coefficient was performed according to the distribution of the data. A p-value <0.05 was considered statistically significant.

## References

[b1] Fang P. . Galanin peptide family as a modulating target for contribution to metabolic syndrome. Gen. Comp. Endocrinol. 179, 115–120 (2012). 22909974 10.1016/j.ygcen.2012.07.029

[b2] Fang P. . Galanin and its receptors: A novel strategy for appetite control and obesity therapy. Peptides 36, 331–339 (2012). 22664322 10.1016/j.peptides.2012.05.016

[b3] Legakis I. N. The role of galanin in metabolic disorders leading to type 2 diabetes mellitus. Drug News Perspect. 18, 173 (2005). 15915218 10.1358/dnp.2005.18.3.892762

[b4] He B. . Beneficial effect of galanin on insulin sensitivity in muscle of type 2 diabetic rats. Physiol. Behav. 103, 284–289 (2011). 21352839 10.1016/j.physbeh.2011.02.023

[b5] Liang Y. . Exercise-induced galanin release facilitated GLUT4 translocation in adipocytes of type 2 diabetic rats. Pharmacol. Biochem. Behav. 100, 554–559 (2012). 22079346 10.1016/j.pbb.2011.10.026

[b6] Kyrkouli S. E., Stanley B. G., Seirafi R. D. & Leibowitz S. F. Stimulation of feeding by galanin: anatomical localization and behavioral specificity of this peptide’s effects in the brain. Peptides 11, 995–1001 (1990). 1704616 10.1016/0196-9781(90)90023-x

[b7] Corwin R. L., Robinson J. K. & Crawley J. N. Galanin antagonists block galanin-induced feeding in the hypothalamus and amygdala of the rat. Eur J Neurosci. 1(5), 1528–1533 (1993). 7506975 10.1111/j.1460-9568.1993.tb00221.x

[b8] Psichas A., Glass L. L., Sharp S. J., Reimann F. & Gribble F. M. Reimann F1, G. F. Galanin inhibits GLP-1 and GIP secretion via the GAL1 receptor in enteroendocrine L and K cells. Br J Pharmacol. Dec 12, (2015). 10.1111/bph.13407 PMC4761093 26661062

[b9] Ahrén B1., Pacini G., Wynick D., Wierup N., Sundler F. Loss-of-function mutation of the galanin gene is associated with perturbed islet function in mice. Endocrinology 145, 3190–3196 (2004). 15044363 10.1210/en.2003-1700

[b10] Poritsanos N. J., Mizuno T. M., Lautatzis M.E. & Vrontakis M. Chronic increase of circulating galanin levels induces obesity and marked alterations in lipid metabolism similar to metabolic syndrome. Int. J. Obes. (Lond). 33, 1381–1389 (2009). 19773738 10.1038/ijo.2009.187

[b11] Bu L., Yao Q., Liu Z., Tang W. & Zou J. & Qu S. Combined galanin with insulin improves insulin sensitivity of diabetic rat muscles. J Endocrinol. 221, 157–165 (2014). 24501381 10.1530/JOE-13-0444

[b12] Invitti C. . Plasma galanin concentrations in obese, normal weight and anorectic women. Int. J. Obes. Relat. Metab. Disord. 19, 347–349 (1995). 7544186

[b13] Meczekalski B. & Warenik-Szymankiewicz A. [The role of galanin in the etiology of obesity in young girls]. Ginekol. Pol. 70, 328–332 (1999). 10462975

[b14] Zhang Z. . Endogenous galanin as a novel biomarker to predict gestational diabetes mellitus. Peptides 54, 186–189 (2014). 24503374 10.1016/j.peptides.2014.01.024

[b15] Zhang Z. . Association between circulating levels of galanin and pre-pregnancy body mass index in patients with gestational diabetes mellitus. Eat. Behav. 19, 57–60 (2015). 26172564 10.1016/j.eatbeh.2015.06.003

[b16] Fang P., Bo P., Shi M., Yu M. & Zhang Z. Circulating galanin levels are increased in patients with gestational diabetes mellitus. Clin. Biochem. 46, 831–833 (2013). 23266294 10.1016/j.clinbiochem.2012.12.013

[b17] Stergiou E. . Effect of Gestational Diabetes and Intrauterine Growth Restriction on the Offspring’s Circulating Galanin at Birth. J. Clin. Endocrinol. Metab. 97, E238–E242 (2012). 22162474 10.1210/jc.2011-1855

[b18] Celi F. . Circulating acylated and total ghrelin and galanin in children with insulin-treated type 1 diabetes: relationship to insulin therapy, metabolic control and pubertal development. Clin. Endocrinol. (Oxf ). 63, 139–145 (2005). 16060906 10.1111/j.1365-2265.2005.02313.x

[b19] Nergiz S. . Circulating galanin and IL-6 concentrations in gestational diabetes mellitus. Gynecol. Endocrinol. 30, 236–240 (2014). 24397394 10.3109/09513590.2013.871519

[b20] Legakis I. N., Mantzouridis T. & Mountokalakis T. Positive correlation of galanin with glucose in healthy volunteers during an oral glucose tolerance test. Horm. Metab. Res. 39, 53–55 (2007). 17226114 10.1055/s-2006-957346

[b21] Gilbey S. G., Stephenson J., O’Halloran D. J., Burrin J. M. & Bloom S. R. High-dose porcine galanin infusion and effect on intravenous glucose tolerance in humans. Diabetes 38, 1114–1116 (1989). 2475378 10.2337/diab.38.9.1114

[b22] Mazziotti G. . Biochemical Evaluation of Patients with Active Acromegaly and Type 2 Diabetes Mellitus: Efficacy and Safety of the Galanin Test. Neuroendocrinology 88, 299–304 (2008). 18617732 10.1159/000144046

[b23] Fang P., Shi M., Zhu Y., Bo P. & Zhang Z. Type 2 diabetes mellitus as a disorder of galanin resistance. Exp. Gerontol. 73, 72–77 (2016). 26585047 10.1016/j.exger.2015.11.007

[b24] Fang P. . Effect of endogenous galanin on glucose transporter 4 expression in cardiac muscle of type 2 diabetic rats. Peptides 62, 159–163 (2014). 25445608 10.1016/j.peptides.2014.10.001

[b25] Guo L. . Galanin antagonist increases insulin resistance by reducing glucose transporter 4 effect in adipocytes of rats. Gen. Comp. Endocrinol. 173, 159–163 (2011). 21664358 10.1016/j.ygcen.2011.05.011

[b26] Jiang L. . Effect of M35, a neuropeptide galanin antagonist on glucose uptake translated by glucose transporter 4 in trained rat skeletal muscle. Neurosci. Lett. 467, 178–181 (2009). 19835935 10.1016/j.neulet.2009.10.034

[b27] Zhang Z. . Intracerebroventricular administration of galanin antagonist sustains insulin resistance in adipocytes of type 2 diabetic trained rats. Mol. Cell. Endocrinol. 361, 213–218 (2012). 22564511 10.1016/j.mce.2012.04.012

[b28] Bauer F. E. . Inhibitory effect of galanin on postprandial gastrointestinal motility and gut hormone release in humans. Gastroenterology 97, 260–264 (1989). 2472997 10.1016/0016-5085(89)90059-0

[b29] Fang P. . The regulative effect of galanin family members on link of energy metabolism and reproduction. Peptides 71, 240–249 (2015). 26188174 10.1016/j.peptides.2015.07.007

[b30] Li R. Y., Song H. D., Shi W. J., Hu S. M., Yang Y. S., Tang J. F. . Galanin inhibits leptin expression and secretion in rat adipose tissue and 3T3-L1 adipocytes. J Mol Endocrinol 33, 11–19 (2004). 15291739 10.1677/jme.0.0330011

[b31] Baranowska B., Wasilewska-Dziubinska E., Radzikowska M., Plonowski A. & Roguski K. Neuropeptide Y, galanin, and leptin release in obese women and in women with anorexia nervosa. Metabolism. 46, 1384–1389 (1997). 9439531 10.1016/s0026-0495(97)90136-0

[b32] Baranowska B., Radzikowska M., Wasilewska-Dziubinska E., Roguski K. & Borowiec M. Disturbed release of gastrointestinal peptides in anorexia nervosa and in obesity. Diabetes, Obes. Metab. 2, 99–103 (2000). 11220530 10.1046/j.1463-1326.2000.00070.x

[b33] Yun R. . PVN galanin increases fat storage and promotes obesity by causing muscle to utilize carbohydrate more than fat. Peptides 26, 2265–2273 (2005). 15893855 10.1016/j.peptides.2005.04.005

[b34] Leibowitz S. F., Akabayashi a. & Wang J. Obesity on a high-fat diet: role of hypothalamic galanin in neurons of the anterior paraventricular nucleus projecting to the median eminence. J. Neurosci. 18, 2709–2719 (1998). 9502828 10.1523/JNEUROSCI.18-07-02709.1998 PMC6793124

[b35] Liang Y. . Exercise-induced galanin release facilitated GLUT4 translocation in adipocytes of type 2 diabetic rats. Pharmacol. Biochem. Behav. 100, 554–559 (2012). 22079346 10.1016/j.pbb.2011.10.026

[b36] Yadav A., Kataria M. A., Saini V. & Yadav A. Role of leptin and adiponectin in insulin resistance. Clin. Chim. Acta 417, 80–84 (2013). 23266767 10.1016/j.cca.2012.12.007

[b37] Pittas A. G., Joseph N. A. & Greenberg A. S. Adipocytokines and insulin resistance. J. Clin. Endocrinol. Metab. 89, 447–452 (2004). 14764746 10.1210/jc.2003-031005

[b38] Kim A. Y. . Obesity-induced DNA hypermethylation of the adiponectin gene mediates insulin resistance. Nat. Commun. 6, 7585 (2015). 26139044 10.1038/ncomms8585 PMC4506505

[b39] Gregor M. F. & Hotamisligil G. S. Inflammatory Mechanisms in Obesity. Annu. Rev. Immunol. 29, 415–445 (2011). 21219177 10.1146/annurev-immunol-031210-101322

[b40] WHO. Obesity and overweight. at http://www.who.int/mediacentre/factsheets/fs311/en/ (2015).

[b41] Garcés M. F. . Longitudinal analysis of maternal serum Follistatin concentration in normal pregnancy and preeclampsia. Clin. Endocrinol. (*Oxf* ). doi:10.1111/cen.12715(2015). 25565002

[b42] Matthews D. R. . Homeostasis model assessment: insulin resistance and beta-cell function from fasting plasma glucose and insulin concentrations in man. Diabetologia 28, 412–419 (1985). 3899825 10.1007/BF00280883

