# Supplementary material for: Serum Galanin Levels in Young Healthy Lean and Obese Non-Diabetic Men during an Oral Glucose Tolerance Test
Source: Sci Rep. 2016 Aug 23;6:31661. doi: 10.1038/srep31661 (PMC4994037; doi:10.1038/srep31661)
Supplement: Supplementary Information [file srep31661-s1.pdf]

Serum Galanin Levels in Young Healthy Lean and Obese Non-Diabetic Men during an Oral Glucose Tolerance Test

Héctor Fabio Sandoval – Alzate[1], Yessica Agudelo – Zapata[1], Angélica María González-Clavijo[1], Natalia E. Poveda[2], Cristian Felipe Espinel - Pachón[2] , Jorge Augusto Escamilla - Castro[2], Heidy Lorena Márquez - Julio[2], Hernando Alvarado – Quintero[2], Fabián Guillermo Rojas – Rodríguez[2], Juan Manuel Arteaga –Díaz[1], Javier Hernando Eslava - Schmalbach[3], Maria Fernanda Garcés - Gutiérrez[2], Maria Vrontakis[4], Justo P. Castaño[5, 7], Raúl Luque[5,7], Carlos Diéguez[6, 7], Rubén Nogueiras[6, 7] and Jorge E. Caminos[2].

[1]Department of Internal Medicine – Division of Endocrinology, [2]Department of Physiology, and [3]Institute of Clinical Investigations, School of Medicine, Universidad Nacional de Colombia, Bogotá, Colombia. [4]Department of Human Anatomy & Cell Science, Faculty of Medicine, University of Manitoba, Winnipeg, Manitoba, Canada. [5]Department of Cell Biology, Physiology, and Immunology, University of Córdoba, Córdoba, Spain; Instituto Maimónides de Investigación Biomédica de Córdoba (IMIBIC), Reina Sofía University Hospital, Córdoba, Spain.[6]Department of Physiology (CIMUS), School of Medicine- Instituto de Investigaciones Sanitarias (IDIS), University of Santiago de Compostela, Santiago de Compostela, Spain. [7]CIBER Fisiopatología de la Obesidad y Nutrición (CIBERobn), Spain.

Supplementary Figure 1. Glucose and insulin levels in healthy, lean men and obese men during an oral glucose tolerance test (OGTT). Blood glucose and plasma insulin concentration in response to oral glucose loading during OGTT in two groups of young men, healthy and obese.

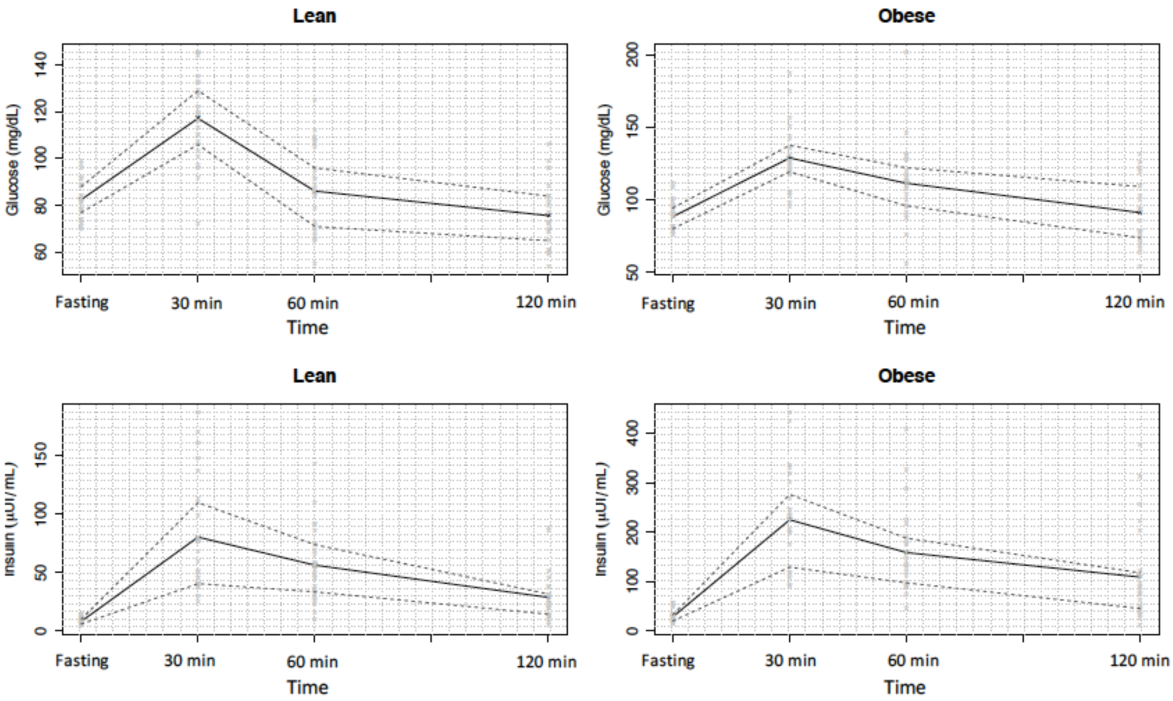

Supplementary Figure 2. Comparison of HOMA-IR index among healthy, lean men and obese men. Box-and-whisker plot with median value, interquartile range, and lower and upper values. The figure shows statistically significant differences in HOMA-IR between healthy lean, men and young, non-diabetic men. *** p<0.001.

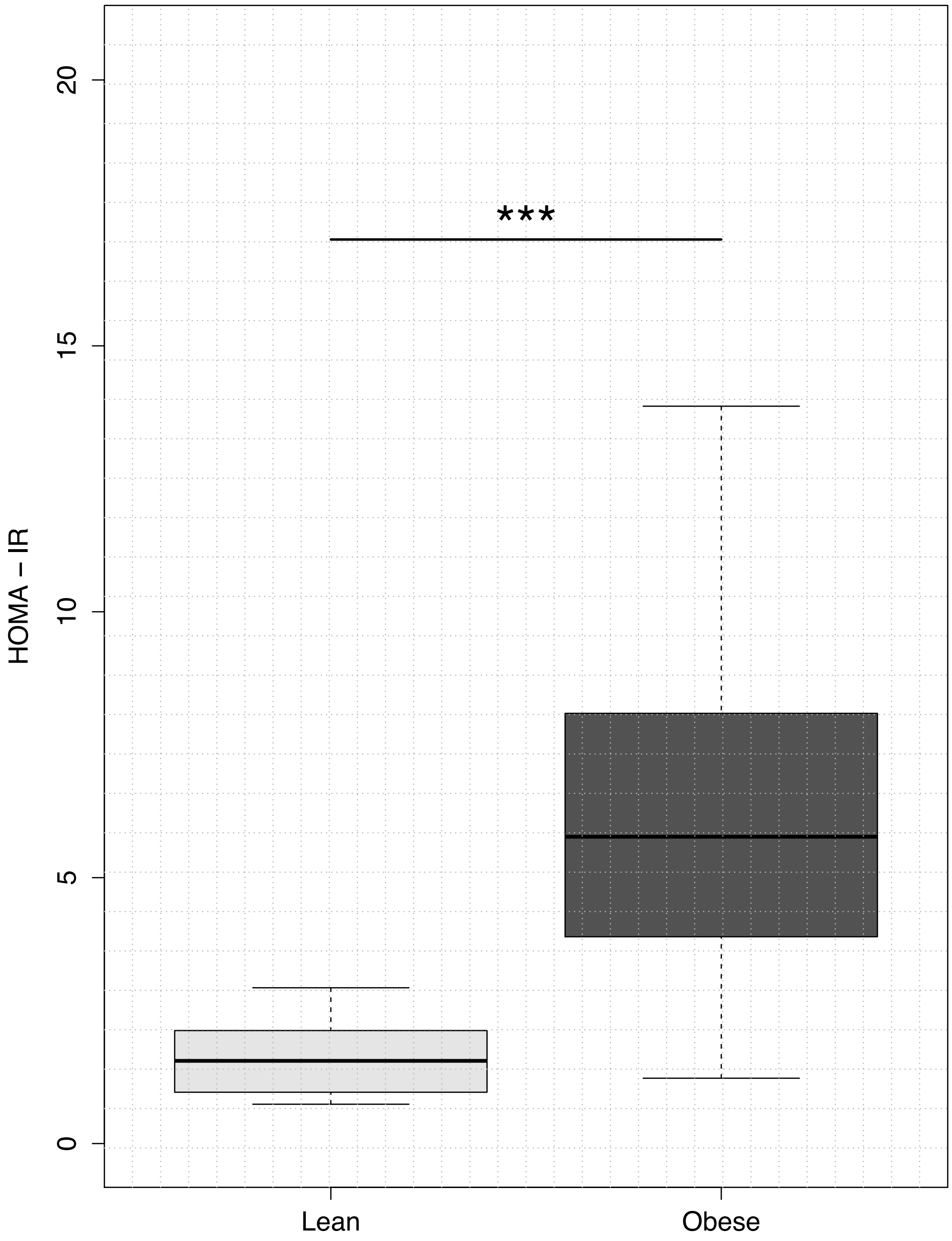

Supplementary Figure 3. Lipid profile parameters in healthy, lean men and obese men. Box-and-whisker plots with median value, interquartile range, and lower and upper values. In the figure are represented the lipid profile parameters measured, total cholesterol, HDL-cholesterol, LDL-cholesterol, VLDL-cholesterol and triglycerides, in two groups of young men (healthy lean and non-diabetic obese). Statistical significance is shown in brackets. Points represent outliers.

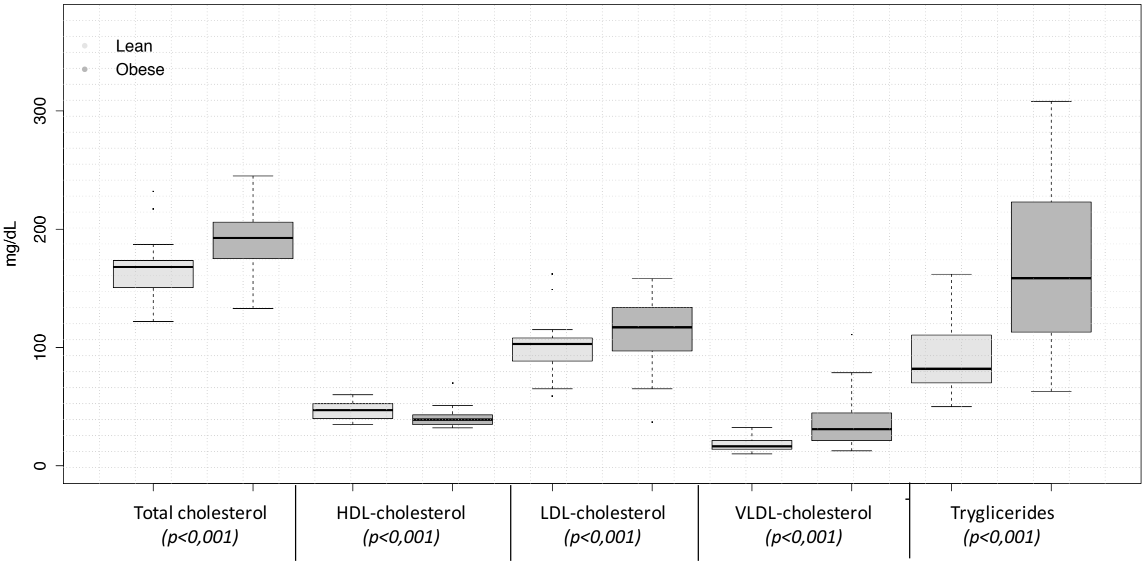

Supplementary Figure 4. Scatterplot of HOMA-IR correlated with serum fasting GAL levels in young, lean and obese non-diabetic men. Positive correlation between HOMA-IR index and fasting GAL levels in a group of 60 young, lean and obese non-diabetic men.

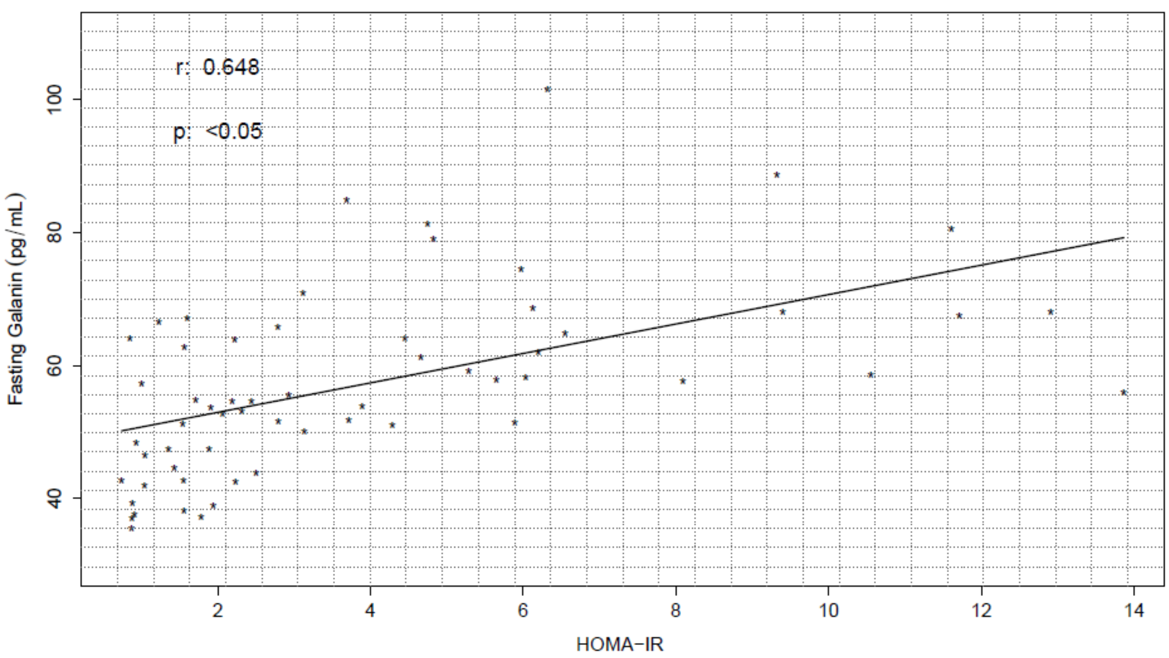

Supplementary Figure 5. Scatterplots of lipid profile parameters correlated with fasting serum GAL levels in young, lean and obese non-diabetic men. Positive correlation between total cholesterol, LDL-cholesterol, triglycerides levels and fasting GAL levels. Negative correlation between HDL-cholesterol levels and fasting GAL levels.

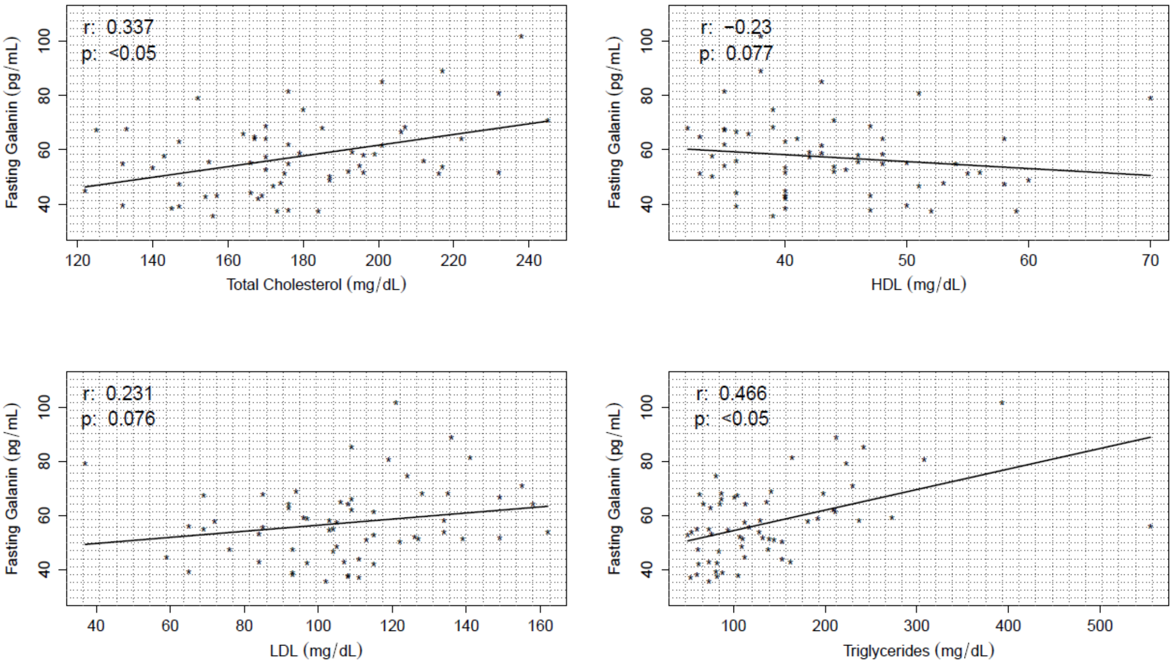

Supplementary Table 1. Multiple regression analysis between GAL and anthropometric, biochemical and serum variables.

**Variable**	**β**	**p-value**
**Fasting GAL**		
BMI, Kg/m^2 *	0.234	0.795
Total fat, % *	-0.291	0.737
Waist circumference, cm	-0.339	0.478
Visceral fat (android), %	0.996	0.128
Fasting Glucose, mg/dL	0.148	0.621
Fasting insulin, µUI/mL	1.935	0.072
HOMA-IR	-6.553	0.155
Total cholesterol, mg/dL	-0.253	0.422
HDL-cholesterol, mg/dL	0.499	0.198
LDL-cholesterol, mg/dL	0.275	0.373
Triglycerides, mg/dL	0.065	0.330
Leptin, pg/mL	-0.000	0.138
Adiponectin, µg/mL	-0.215	0.793
**GAL 30 min**		
Glucose 30 min, mg/dL	0.052	0.544
Insulin 30 min, µUI/mL	0.098	<0.001
**GAL 60 min**		
Glucose 60 min, mg/dL	-0.088	0.411
Insulin 60 min, µUI/mL	0.178	<0.001
**GAL 120 min**		
Glucose 120 min, mg/dL	-0.061	0.656
Insulin 120 min, µUI/mL	0.189	<0.001

A p-value <0.05 was considered statistically significant.
